# Rabies Outbreak in the Urban Area of Delhi: An Investigation Report and One Health Perspective for Outbreak Management

**DOI:** 10.3390/idr14060102

**Published:** 2022-12-14

**Authors:** Monil Singhai, Vishesh Sood, Girraj Singh, Cordelia Siddiqui, Tushar Nale, Prabhakar Jha, Priyanka Yadav, Rekha Jaiswal, Manju Bala, Sujeet K. Singh, Simmi Tiwari

**Affiliations:** 1 Centre for Arboviral and Zoonotic Diseases, National Centre for Disease Control, New Delhi 110054, India; 2 Division of Zoonotic Diseases Program, National Centre for Disease Control, New Delhi 110054, India

**Keywords:** rabies, outbreak, multidisciplinary team, one health approach

## Abstract

Rabies is a global problem and is endemic in India. Rabies cases occur throughout the year, and the majority of cases are associated with dog bites. We report a rabies outbreak investigation in an urban area of Delhi conducted by our multidisciplinary team, and its role in proactively controlling a rabies outbreak by concerted efforts and timely advice to various stakeholders using a “One Health Approach.” A veterinary care NGO from Delhi picked up a suspected rabid stray dog and submitted a brain sample for diagnosis of rabies, as they had received information from a resident of the locality about an unprovoked animal bite incident involving a girl (category III bite) and close contact of two more stray dogs living in the vicinity of the suspected rabid dog. The laboratory diagnosis of rabies in the suspected dog brain sample was confirmed by using Fluorescence Antibody Test (FAT). A multi-expert investigation team with expertise in medicine, microbiology, veterinary sciences, laboratory diagnosis, and public health was constituted to investigate the outbreak. The timely, adequate, and appropriate anti-rabies management initiated for the animal bite victims in this incident could prevent rabies. Proactive involvement of multiple stakeholders and knowledge attributes and practice of local residents could prevent human rabies. As there were no further reports of dog bites from the area, the chain of rabies transmission in that area could be controlled. The presented work is a classical case scenario where concerted efforts of all stakeholders achieved effective control and prevention of rabies by adopting the “One Health approach”.

## 1. Introduction

Rabies virus is a neurotropic virus usually presenting with acute neurological symptoms. Rabies is a global problem and is endemic in India. Rabies cases occur throughout the year, and the majority of cases are associated with dog bites. It has also been reported in a study from the anti-rabies clinic of the tertiary care hospital in Delhi that the majority (91.0%) of animal bite cases belonged to category three, and were due to dog bites from the years 2010 to 2018 [1]. India accounts for the most deaths in Asia (59.9% of human rabies deaths) and globally (35% of human rabies deaths). More than 95% of human rabies cases are transmitted by dogs [2].

Rabies cases in dogs and wildlife animals such as deer have been reported in the past from the National Zoological Park in Delhi. Mass mortality of spotted deer was recorded at the National Zoological Park, New Delhi, India, in 2016, due to a rabies outbreak that was laboratory-confirmed by direct fluorescent antibody test (FAT) and nucleic acid amplification test (NAAT) followed by sequencing in our previous study [3,4]. 

National reference laboratory for rabies at the Centre for Arboviral and Zoonotic Diseases (CAZD), National Centre for Disease Control (NCDC), is a part of the World Health Organization collaborating centre for rabies epidemiology. It routinely provides referral diagnostic services for rabies in animal bite victims and rabies in dogs/other animals, and contributes to epidemiological and knowledge generation activities pertaining to rabies. We report a rabies outbreak investigation in an urban area of Delhi conducted by our multidisciplinary team, and its role in proactively controlling a rabies outbreak by concerted efforts and timely advice to various stakeholders using a “one health approach”.

## 2. Materials and Methods

### 2.1. Incident Details

A veterinary care Non Government Organization (NGO) from Delhi, picked up an alive suspected rabid stray dog in Delhi on 20 February 2022 for diagnosis of rabies, as they received information from a resident of the locality about an unprovoked animal bite incident involving a girl (category III bite), and close contact of two more stray dogs living in the vicinity of the suspected rabid dog.

### 2.2. Laboratory Diagnosis of Rabies

The suspected rabid dog died during the first day of the observation period and the cerebellum part of the brain was submitted to the CAZD, NCDC, New Delhi, for laboratory diagnosis.

The laboratory diagnosis of rabies in the suspected dog sample was confirmedby detecting rabies virus antigen on brain impression smears by DFAT, using anti-rabies nucleocapsid fluoroisothiocynate conjugate (BioRad GmbH, Munchen, Germany) performed as per the manufacturer’s recommendation. 

Other laboratory test done for Rabies in brain sample were detecting rabies-specific RNA in the sample by pan-lyssavirus nested RT-PCR protocol as per the WHO Manual on Laboratory Techniques in Rabies [5] and Rapid Diagnostic test (RDT) (Anigen Rapid Rabies Ag test kit (Bionote, Hwaseong-si, Republic of Korea). For RT-PCR, the RNA was extracted from 10% homogenate of the brain sample in PBS. 

### 2.3. Outbreak Investigation

For the outbreak investigation of the incident, a framework based on the recommendations of Goodman et al. [6] was used. Briefly, the steps followed were as follows:

1. Preparation for the field visit. On laboratory confirmation of rabies in a dog sample, all required administrative approvals were obtained from the Director, National Centre for Disease Control, New Delhi, to undertake a field investigation to establish the extent of rabies exposure in animals and humans in the vicinity. A multi-expert investigation team with expertise in medicine, microbiology, veterinary sciences, laboratory diagnosis, and public health was constituted;
2. Verification of the laboratory diagnosis. The team of experts verified the laboratory confirmation of rabies;
3. Orientation of data in terms of time, place, and person. The antecedents of the laboratory-confirmed rabid dog were established. The Veterinary NGO was approached to identify the exact location from where the dog was picked up. The team then prepared a plan to visit the place and interview the locals to understand the extent of rabies exposure to humans and animals. The investigation team visited the site and established that one girl had significant exposure to the laboratory-confirmed rabid dog with a category three bite. An inquiry was made to establish if there were any other cases of significant human exposure in that area during the incident;
4. Evaluation and implementation of control and preventive measures. The investigation team inquired about the rabies control practices for animal bite victims in the area. The exposed girl was immediately taken to an anti-rabies clinic. The public was informed about the importance of rabies prevention and control using the material prepared in the native language;
5. Communication. A detailed report with recommendations was shared with all the relevant shareholders for further public health actions.

## 3. Results

### 3.1. Laboratory Confirmation of Rabies in the Dog

**Figure 1:**
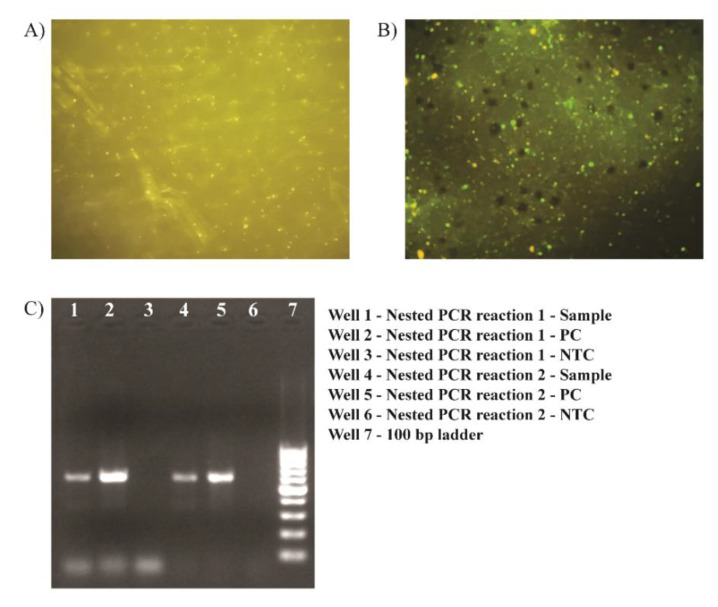
Fluorescence micrograph (400× magnification) of direct Fluorescence Antibody Test (FAT) performed on impression smear of the brain sample of (**A**) normal mouse, and (**B**) cerebellum of the suspected rabid stray dog. (**C**) Documented gel image of nested RT-PCR for pan-lyssavirus RNA showing characteristic bands at ~606 bp (first round) and ~582 bp (second round) with the valid positive control (PC) and no template controls (NTC).

The cerebellum part of the brain of the suspected rabid dog was submitted to CAZD on 21 February 2022. The sample tested positive for rabies virus antigen (by FAT and RDT), and rabies virus nucleic acid (by RT-PCR). Based on the test results, the case was classified as a laboratory-confirmed case of dog rabies (Figure 1).

### 3.2. Site Visit and Interview of the Animal Bite Victim

Based on the available information, an online review of the site revealed that the place of incidence was a slum cluster surrounded by gated communities. The gated communities had limited access to stray dogs from the street. Therefore, the site visit and interview were limited to the slum cluster area. The slum cluster had 40 households limited to approximately 0.2 square kilometer area [7]. For the interview, various people who could have been at risk of exposure were selected, including the person who reported the incident, the mother of the animal bite victim, and animal caregivers in the area. Some other people were randomly selected and interviewed to ascertain the extent of the outbreak in stray dogs of the area or information regarding human exposure other than that reported.

**Figure 2:**
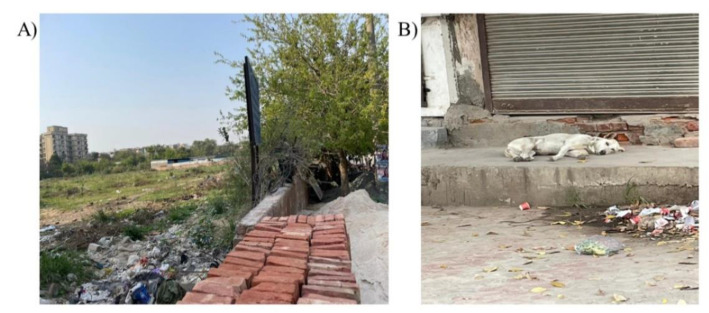
(**A**) Garbage dump in the empty land near the incident site. (**B**) The stray dog which was exposed to the laboratory-confirmed rabid dog during the incident.

On interviewing, residents informed that the witness of the incident had a shop near the incident site. The area was clean. However, an empty plot with garbage and waste was found near the incident site (Figure 2).

**Figure 3:**
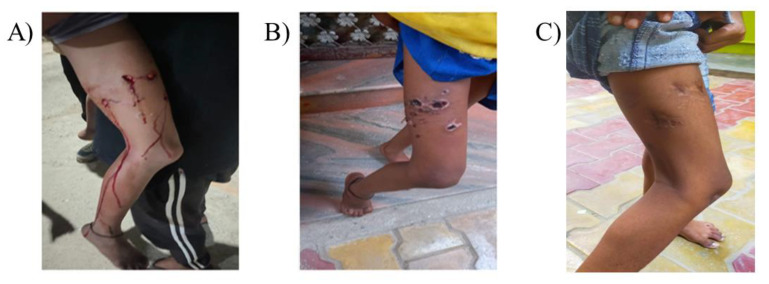
(**A**) Multiple deep wounds with profuse bleeding dated 20 February 2022, on girl’s right thigh after bite from the suspected rabid dog, (**B**) Wound in healing stage dated 3 March 2022, after timely, adequate, and appropriate anti-rabies management initiated, and (**C**) wound completely healed (dated 25 August 2022).

The resident who witnessed the incident recalled a significant unprovoked exposure (category III bite) in one case of a 7-year-old girl on the evening of 20 February 2022. The interviewee took the team to the animal bite victim’s (ABV) house. The team visited this ABV’s home and interviewed the girl’s mother. The mother noted that there were multiple deep wounds with profuse bleeding on the girl’s right thigh (Figure 3).

ABV’s mother immediately cleaned the wound with Dettol (an over-the-counter commercial antiseptic available in Indian markets) and water and took her to a tertiary care hospital (Ram Manohar Lohia Hospital) in Delhi. She was given anti-rabies serum, tetanus toxoid, and anti-rabies vaccine as per the prescription dated 20 February 2022. The team examined the bite site and observed that the wound was healthy with scabs and granulation tissue. The mother was advised to complete the full vaccination course and visit the CAZD division for anti-rabies antibody titer estimation after 15 days of completion of post-exposure prophylaxis if desired.

### 3.3. Information, Education, and Communication (IEC) Activity Carried out for Local Residents of Areas for Awareness

A group of around 20 residents from the locality who were witnessing the interview were targeted for IEC activities. The residents were informed to have timely, adequate, and appropriate anti-rabies management in case of any other significant exposure from an animal bite in the locality.

They were also informed about steps and points to remember for animal bite management and the importance of wound washing with soap and water. IEC material was handed over to the Anganwadi helper (person overlooking the government run childcare centers in India) in that area. 

The team could locate three more dogs in that vicinity. The residents and animal care givers were also informed to take care of anti-rabies vaccination/birth control in street dogs, keep a note on other dogs who had been in close contact with the suspected rabid dog for behavior change, and were advised to contact Veterinary Hospital, opposite Bhagat Chandra Hospital, below Palam flyover, Palam Dabri Road, Palam, New Delhi for further necessary action if any required.

### 3.4. Visit to the Veterinary Hospital, New Delhi

A visit was made to the Deputy Director, Southwest Delhi, Animal Husbandry Department, Govt of Delhi, debriefing the whole incident to him. The team shared the relevant documents with the authorities. Authorities were also requested to coordinate with South Delhi Municipal Corporation, Delhi, for animal birth control and mass vaccination to control dog-mediated rabies in that area.

### 3.5. Recommendations

1. Anti-rabies vaccination camp/animal bite management of other animals with exposure/animal birth control activities:
  - For vaccination in India and several Asian countries, many commercially available rabies vaccines are licensed for post-exposure prophylaxis in animals and may be given as per the 5-dose intramuscular schedule for humans (0, 3, 7, 14, and 28 days) [8];
  - As there is no consensus worldwide regarding the isolation/observation period of animals exposed to a suspected rabid dog, the observation period for exposed dogs may vary based on epidemiological information such as previous vaccination status, type of injury, or the fate of the biting animal. The treating veterinarian may recommend euthanasia or isolation for a specific observation period of such animals. The treating veterinarian must follow all the regulatory guidelines for institutional isolation or euthanasia in case of high-risk exposure. Relevant international guidelines may be used as a reference at the veterinarian’s discretion for animal observation or isolation periods. The recommended period of isolation for dogs in cases of significant exposure to a human being is 10 days [9]. After significant exposure to a laboratory-confirmed rabid dog, a vaccinated dog may be observed for 45 days, and an unvaccinated dog may be observed for 4–6 months [10,11];
  - A target of vaccinating 70% of the canine population in the area may be achieved to minimize dog-mediated rabies [12]. For animal birth control activities, a female-centric sterilization approach involving at least 70% of female dogs and 30% of male dogs [13] based on guidelines provided in the *Animal Birth Control (Dogs) Amendment Rules 2010* [14] under the *Prevention of Cruelty to Animals Act, 1960* [15].

In view of the above, the Veterinary/Municipality department may immediately enhance the anti-rabies vaccination camp/animal bite management of other animals with exposure/animal birth control activities if changes in the behavior of stray dogs or an increase in animal bites are reported in the area.

2. IEC Activities
  - Multi stakeholders team including human health authorities, veterinary authorities, sanitation and municipality authorities, animal activists, public and civic bodies, NGOs etc., may be formed to carry out an IEC campaign for resident to address the knowledge gaps which may help to minimize such animal-to-human and animal-to-animal bites in future.
  - In areas where animal bite incidences are being increasingly reported in humans, the IEC campaign may focus on the importance of wound washing with soap and water followed by timely and appropriate management of category III animal bite victims by postexposure prophylaxis measures (anti-rabies serum, tetanus toxoid, and anti-rabies vaccine).
3. Environmental cleanliness and solid waste management (garbage disposal)
  - Residents may inform the health authorities/animal welfare authorities/sanitation and municipality if incidences of animal-to-animal bites or stray dog menaces increase in the area. One of the main reasons for the increase in stray dog numbers is the inappropriate waste disposal methods, especially the remaining food to which stray dogs naturally get attracted. Environmental cleanliness and solid waste management (garbage disposal) should be enhanced, which is a shared responsibility of citizens and the Municipality Department.
4. Monitoring the efficacy of the anti-rabies vaccine
  - The parenteral anti-rabies vaccines available commercially for humans and animals are quite efficacious if administered as per schedule. The anti-rabies titer estimation for humans and canines who have received post-exposure prophylaxis is usually not recommended if the vaccination schedule is strictly followed.
  - The treating physician or veterinarian may recommend anti-rabies antibody titer estimation given a deviation of schedule, default in route or dose prescribed for vaccination, immune status of the vaccinated individual (immune tolerant or immunocompromised), or probability of repeated occupational exposures.

In India, an existing network of referral government laboratories performs anti-rabies antibody titer estimation either freely or on nominal charges [9]. Testing facilities are advised to monitor the efficacy of vaccination and practices of animal bite management in health care settings from time to time for identifying the knowledge gaps, especially if frequent departures from the recommended schedules are observed.

5. Pre-exposure and Post-exposure Prophylaxis
  - People in the area who are at risk of repeated exposure, for example, animal caregivers providing food to stray dogs, sanitary workers, etc., are advised to ensure that they receive pre-exposure anti-rabies prophylaxis as per the national guidelines.
  - Health authorities should ensure uninterrupted and adequate supply of Anti-rabies vaccine and serum at all times in designated Anti-rabies clinics and health care facilities.

## 4. Discussion

This manuscript reports a rabies outbreak in an urban setting in Delhi, India. A suspected rabid dog had bitten a child. The dog was picked up by an NGO and the dog died during the first day of the observation period. The brain sample of the dog was submitted for laboratory confirmation to CAZD, NCDC, Delhi. An outbreak investigation was initiated after the laboratory confirmation of rabies to assess the spread of rabies in and around the area. This work highlights the role of proactive involvement of multi-stakeholders and knowledge attributes practices of residents in preventing human rabies. The timely, adequate, and appropriate anti-rabies management initiated for ABV in this incident prevented rabies. It has been reported that animal bite cases are increasing over the years and the most commonly reported animal bites in Delhi are dog bites. Therefore, there is a need to implement IEC activities and carry out awareness programs among people regarding animal bite management [1]. The “One Health Approach” involving inter-sectoral collaboration among animal, human, and environmental health can efficiently help to control and prevent rabies [16].

Coordination and communication of health professionals play an essential role in the overall success of strategies for controlling and preventing rabies [17,18,19]. Active surveillance generates the primary data that would contribute to figuring out the current disease burden [20]. It is important to carry out animal birth control activities, mass vaccination of dogs in particular geographical areas, and breaking the transmission chain when confirmed rabies is being reported from a particular area. The outbreak investigation, in this case, was carried out as soon as laboratory methods confirmed the suspected rabies in the dog. This resulted in creating awareness at the incident site and sensitizing people about pre-exposure/post-exposure prophylaxis aspects of anti-rabies vaccination, which is essential for all individuals with a significant probability of exposure to dogs such as rag pickers, school-going children, animal care workers in resident welfare associations, etc. 

Apart from technical aspects of inter-sectoral cooperation, the successful application of “One Health” approaches also requires the development of nontechnical expertise and collaborations such as advocacy, knowledge translation, evidence-based decision making, and capacity development [21]. Throughout the outbreak investigation, the focus remained on providing the above through IEC activity for rabies control and management. 

As humans, domestic animals, and wildlife territories are in close proximity, coordination of all sectors is equally essential to execute a successful plan. It has been found that mass vaccination of canines (both stray and pet) at a common site and a combined approach of vaccination and dog population management are important strategies in rabies control [22]. Therefore, the major emphasis of the team was to sensitize the Animal Husbandry Department of Delhi to take appropriate measures, such as a mass vaccination campaign against rabies in and around the area of incidence and animal birth control activities if required. 

As there were no further reports of suspected rabies from the area, the chain of rabies transmission in dogs could be controlled. The presented work is a classic case scenario where concerted efforts by all stakeholders achieved effective control and prevention of rabies by adopting the “one health approach”. 

## Data Availability

Not applicable.
